# Personality, self-rated health, and cognition in centenarians: Do personality and self-rated health relate to cognitive function in advanced age?

**DOI:** 10.18632/aging.100545

**Published:** 2013-03-23

**Authors:** Kaori Kato, Richard Zweig, Clyde B. Schechter, Joe Verghese, Nir Barzilai, Gil Atzmon

**Affiliations:** ^1^ Ferkauf Graduate School of Psychology, Bronx, NY 10461, USA; ^2^ Department of Family and Social Medicine and Epidemiology and Population Health, Albert Einstein College of Medicine, Bronx, NY 10461, USA; ^3^ Departmen of Neurology, Albert Einstein College of Medicine, Bronx, NY 10461, USA; ^4^ Departmen of Medicine, Albert Einstein College of Medicine, Bronx, NY 10461, USA; ^5^ Genetics, Albert Einstein College of Medicine, Bronx, NY 10461, USA

**Keywords:** personality, cognition, self-rated health, centenarian, aging

## Abstract

Personality and self-rated health have been linked previously to cognitive outcome in late life. However, these associations have not been shown among the oldest old. This study examined relationships between personality, self-rated health, and cognitive function in a selected sample of Ashkenazi Jewish centenarians (n = 68, 59% female) aged 95 to 106 who lived independently in the community. Personality was measured using the Personality Outlook Profile Scale (POPS), a brief measure that was validated in this population. Self-rated health was assessed by participants’ subjective rating of their present health, and Mini-Mental Status Examination was used to determine cognitive function. Results showed positive associations of the Positive Attitude Towards Life domain of the POPS and self-rated health with participants’ current cognitive function. These associations remained significant even after adjusting for the effects of participants'age, gender, marital status, education, and history of medical illnesses. Further exploratory analysis using structural equations modeling showed significant associations among the three variables, but demonstrated a borderline significant level of mediating effect of personality on the relationship between self-rated health and cognition. These results reemphasized the independent roles of personality and self-rated health on centenarians’ cognitive outcomes. Future studies will further elucidate the impact of personality and self-rated health on cognitive outcomes in the oldest old.

## INTRODUCTION

Centenarians have been suggested to be an example of successful aging due to their delay or escape of major illnesses [1]. Many centenarians have been reported to delay the onset of cognitive impairment until their 90s [2]. More specifically, super-centenarians have shown the longer delay in their age of medical illnesses onset as well as physical and cognitive declines than other oldest old individuals. [3]. While the prevalence rates of dementia are as high as 50% among centenarians [4], a subgroup of those individuals seems to sustain intact cognition. Although numerous studies have attempted to identify risk and protective factors related to cognitive function as well as genotypes associated with cognitive reservation[5-7],components associated with preserved cognitive function in the centenarian population remain unclear.

Among the contributors to successful aging and longevity, personality has been particularly associated with favorable health outcomes, longevity/mortality, and cognitive function in late life [8-10]. While some studies argue that some personality traits arise exclusively from biological/genetic factors and are stable and continuous across the lifespan [11], others have proposed that environmental factors may play an important role [12] and that personality may be malleable depending on the gene-environmental interactions [13]. Personality has also been reported to change in late life in response to cognitive decline or neurodegenerative processes [14]. Further, developmental studies suggest some positive personality changes associated with aging including improved emotional regulation, tolerance, and maturity [15], and such positive changes may contribute to psychological resilience and facilitate adjustment to age-associated physical/social changes [16].

The Five Factor Model proposes that five universal traits that explain personality variability including neuroticism/emotional stability, extraversion, conscientiousness, openness to experience, and agreeableness [14]. Several cross-sectional studies have identified that centenarians and their offspring share prototypical patterns of these traits (e.g., high extraversion and conscientiousness; low neuroticism) [12, 17, 18]. Our recent study also demonstrated that centenarians had higher levels of emotional stability and conscientiousness than the US adult population [19]. In addition, a number of longitudinal studies have reported that these traits predict longevity [20-22] as well as cognitive outcomes [23]. A recent study with older adults reported that high neuroticism and extraversion as well as low openness predicted poor cognitive function over a seven-year period and that older adults with high conscientiousness showed a slow rate of cognitive decline while those with high neuroticism had a steep rate of cognitive decline [24]. Several hypothesized pathways have been suggested to elucidate the link between personality and cognitive function. Personality may indirectly influence cognitive outcomes through its adverse impact on biological systems (e.g., hypothalamic-pituitary adrenal axis and glucocorticoids) or through salutary processes such as emotion regulation and beneficial lifestyles/activities (e.g., physical activity and dietary habits) [25-30].

Another determinant that affects cognitive function in late life is self-rated health [31]. In this line, a prospective study with community-dwelling older adults reported that older adults with poor to fair self-rated health had a higher incidence of both Alzheimer's and vascular dementia compare to those with good self-rated health [32]. Similarly, poor self-rated health has been reported to predict cardiovascular and cerebrovascular events as well as all-cause dementia [33]. Notably, self-rated health has also been linked to certain personality traits including neuroticism, extraversion, and conscientiousness [34-36]. Personality appears to play an important role in older adults’ subjective perceptions of their health status, and it can be speculated that personality may partially explain or mediate the relationship between self-rated health and cognitive function, as maladaptive personality styles may cause individuals to distort perceptions about their health [37].

The current study investigated the role of personality and self-rated health in cognitive function through assessing cross-sectional relationships among personality, self-rated health, and cognitive function in a group of Ashkenazi Jewish centenarians who lived independently in the community with less than a part-time home/nursing care. This group was thought to be a model of successfully aging. Personality was assessed by the Personality Outlook Profile Scale (POPS) [19], a brief measure of personality which represents personality phenotypes which may contribute to successful aging. POPS consists of two personality characteristics, Positive Attitude Towards Life (PATL: optimism, easygoing, laughter, and introversion/outgoing) and Emotional Expression (EE: expressing emotions openly and not bottling up emotions). This measure has been validated with aspects of the Five Factor Model including neuroticism, extraversion, and conscientiousness as well as dispositional optimism of the Life Orientation Test-Revised (LOT-R) [12, 19, 38]. In the present study, it was hypothesized that personality as assessed by the POPS would be positively associated with self-rated health and global cognitive function as assessed by MMSE and that self-rated health would also be positively associated with cognitive function in Ashkenazi Jewish centenarians who were living independently in the community.

## RESULTS

Demographic, personality, behavioral, and clinical characteristics of the sample (N = 68) are presented in Table 1. Participants had a mean age of 97.88 (SD = 2.74) and were predominantly female (59%). 72% of the participants were widowed, 19% were married/cohabitating, 6% were never married, and 3% were separated or divorced. Years of education ranged from 3 (1.5%) to 24 years (1.5%) with a mean level of 14.41 years (SD = 3.89). The MMSE mean score was 24.16 (SD = 9.89). 17.6% scored below a cut off score of 21 on the MMSE (an age-adjusted cut off score for centenarians [39-41]) for clinically significant cognitive impairment; this indicates that 82.4% of the sample appeared cognitively intact.

**Table 1:** Demographic and Clinical Characteristics of Centenarians (N = 68)

Variables		Mean ±SD	N(%)
Age		97.88±2.74	
Gender	Males		28(41)
	Females		40(59)
Marital Status	Married/Cohabitating		13(19)
	Separated/Divorced		2(3)
	Widowed		29(72)
	Never Married		4(6)
Years of Education		14.41±3.89	
POPS – Personality Domains	PATL (z-score composite)	.13±.69	
	Optimism	3.35±1.23	
	Easygoing	3.41±1.19	
	Laughter	3.49±.68	
	Introversion/Outgoing	3.84±1.09	
	EE (z-score composite)	.02±.77	
	Bottle Up Emotions	2.65±.93	
	Express Feelings Openly	2.59±.82	
MMSE		24.16±9.89	
Self-Rated Health		2.65±.94	

*Note*. MMSE = Mini Mental Status Exam; POPS = Personality Outlook Profile Scale; PATL = Positive Attitude Towards Life; EE = Emotional Expression

### Association between the POPS and MMSE

The results of the multiple regression analyses (Table 2) showed that PATL accounted for a significant proportion of the MMSE score variability (β= .36, p < .01). PATL remained significantly associated with MMSE following hierarchical regression analysis (β= .29, p < .05), even after adjusting for age, gender, marital status, education, and history of medical illnesses (Table 3). However, there were significant effects on MMSE by age (β = −.32, p < .01). There was no significant relationship between EE and MMSE. Further, the results showed that self-rated health accounted for a significant proportion of the MMSE score variability (β = .34, p < .01). Self-rated health also remained significantly associated with MMSE following hierarchical regression analysis (β= .32, p < .05), even after adjusting for age, gender, marital status, education, and history of medical illnesses (Table 4). However, there were significant effects on MMSE by age (β= −.32, p < .01) in this model as well.

**Table 2:** Summary of Regression Analysis for POPS and Self-Rated Health Predicting MMSE

IV	R	R^2^	B	SEB	β
PATL	.36	.13**	5.22	1.64	.36**
EE	.07	.00	.85	1.56	.07
Self-Rated Health	.34	.11**	3.51	1.21	.34**

*Note*. POPS = Personality Outlook Profile Scale; IV = Independent Variable; PATL = Positive Attitude Towards Life; EE = Emotional Expression; MMSE = Mini Mental Status Exam.

* p < .05

** p < .01

*** p < .001

**Table 3:** Summary of Hierarchical Regression Analysis for PATL Predicting MMSE after Adjusting for Demographic Variables and History of Medical Illnesses

IV	β	R^2^	R^2^_change_
Step 1		.17*	
Age	−.32**		
Gender	−.05		
Marital Status	.11		
Education	.16		
Step 2		.17	.00
Age	−.32*		
Gender	−.05		
Marital Status	.11		
Education	.16		
History of Medical Illnesses	−.02		
Step 3		.24	.07*
Age	−.21		
Gender	−.06		
Marital Status	.14		
Education	.18		
History of Medical Illnesses	.01		
PATL	.29*		

*Note*. IV = Independent Variable; PATL = Positive Attitude Towards Life; MMSE = Mini Mental Status Exam.

* p < .05

** p < .01

*** p < .001

**Table 4:** Summary of Hierarchical Regression Analysis for Self-Rated Health Predicting MMSE after Adjusting for Demographic Variables and History of Medical Illnesses

IV	β	R^2^	R^2^_change_
Step 1		.17*	
Age	−.32**		
Gender	−.05		
Marital Status	.11		
Education	.16		
Step 2		.17	.00
Age	−.32*		
Gender	−.05		
Marital Status	.11		
Education	.16		
History of Medical Illnesses	.02		
Step 3		.25	.08*
Age	−.31*		
Gender	.08		
Marital Status	.08		
Education	.12		
History of Medical Illnesses	.04		
Self-Rated Health	.32*		

*Note*. IV = Independent Variable; MMSE = Mini Mental Status Exam.

* p < .05

** p < .01

*** p < .001

### Exploratory Analyses

We conducted further exploratory analyses to test a mediation model in which PATL mediates the relationship between self-rated health and MMSE. This model was tested to clarify the relationships among these variables. Personality was thought to partially mediate the relationship between self-rated health and cognition as maladaptive personality may lead to distorted perceptions about one's health status [37]. Prior to testing the mediation model, the patterns of correlations provided preliminary support for the associations among these variables. The results of correlation analyses showed that self-rated health was positively associated with MMSE (r = .36, p < .01) and PATL (r = .38, p < .01). PATL was also positively associated with MMSE (r = .34, p < .01). Subsequently, structural equation modeling (SEM) was used to test a mediation model in which PATL mediates the relationship between self-rated health and MMSE. To test this model, the total, direct, and indirect effects among variables were computed. A total effect represents the association between self-rated health and MMSE. While a direct effect represents the direct association between self-rated health and MMSE controlling for a mediating variable (PATL), an indirect effect corresponds to the mediating effect of PATL in that relationship. The results of SEM showed that the total effect of self-rated health on MMSE as expressed by the path coefficient was 3.51 (95% CI 1.17 – 5.85), p < .01. This total effect was decomposed into a direct effect of self-rated health on MMSE with a path coefficient of 2.41 (95% CI -.02 -4.84), p = .052 and an indirect effect of PATL on the relationship between self-rated health and MMSE with a path coefficient of 1.10 (95% CI -.02-2.22), p = .055, suggesting a borderline level of the statistical significance.

## DISCUSSION

The present study investigated the cross-sectional relationshipsof centenarians’ personality andhealth-rated health to cognitive function in a genetically and environmentally homogenous group of Ashkenazi Jewish centenarians. We demonstrated small but significant associationsof the POPS-PATL and self-rated healthwith centenarians’ cognitive function. These associations remained significant even after adjusting for the effects of age, gender, marital status, education, and history of medical illnesses, suggesting that these were small yet meaningful effects. Further exploratory analyses to test a mediation model showed a borderline significant indirect effect of PATL on the relationship between self-rated health and MMSE.

Findings of the present study are generally consistent with previous studies which support the relationships of personality and self-rated health to cognitive function in the general older adult populations [23, 24, 31-36]. In our previous study, PATL was tied to aspects of extraversion, conscientiousness, and emotional stability/neuroticism of the Five Factor Model as well as dispositional optimism[14, 19, 38]. These personality traits have been consistently linked to positive health outcome and cognitive preservation as well as longevity/mortality [9, 18, 28, 42]. Several studies have suggested that personality may influence important health outcomes in late life through various pathways [25-30]. Although the findings of the current study showed a borderline significant level of a mediating effect of personality in the relationship between self-rated health and cognitive function, there may be other mechanisms which can explain this association. Further, some argue that psychological symptoms (e.g., depression) may affect self-rated health [43], and depression may explain the relationship of personality to both self-rated health and cognition [25, 44]. The current study has a number of limitations.A small sample size may have contributed to the borderline significance of the findings from the test of a mediation model. The study's design was also limited to cross-sectional analyses; thus, findings were unable to establish a causal/sequential relationship between variables. As some previous studies suggest long-term effects of personality on cognitive outcome, a longitudinal design might provide a different perspective to this question. Additionally, responses to the personality items were provided by centenarians with their adult children's assistanceto increase the reliability of participants’ responses. Although sufficient levels of agreement between self- and informant-reports of personality measures have been demonstrated in other studies[45, 46], and our study targeted the oldest old participants to enhance the reliability of their self-reports, we cannot be certain of the influence of informant-reports on our data. The use of a genetically and environmentally homogeneous group may reduce the generalizability of this study's findings. However, the Ashkenazi Jews are not expected to have more longevity than other groups [47] and be resistant to common environmental health risks [48]. This implies that these results may well be relevant to other population. Lastly, this study may lack sufficient coverage of cognitive domains since the MMSE was the only measure used to assess cognition. The results of the current study need to be replicated with the use of more granular measures of both personality and cognition in larger samples of the oldest old populations.

### Conclusions and Directions for Future Research

In summary, the purpose of this study was to examine cross-sectional associations of personality and self-rated health to cognitive functioningin Ashkenazi Jewish centenarians who were living independently in the community. This study adds to a growing body of knowledge which suggests that personality as well as self-rated health may play important roles in cognitive function in advanced age. Although personality was also associated with self-rated health, the mechanisms which explain these relationships among personality, self-rated health, and cognition, which may also have therapeutic implications for prevention and/or treatment for cognitive decline, remain unclear. Thus, further investigation of long-term effects of personality and self-rated health on cognitive functioning is warranted.

## METHODS

### Participants

241 Ashkenazi Jewish centenarians (75% female, ages 95-107y) were recruited to the study. Based on participants’ living arrangements and levels of homecare, 166 participants residing in a skilled-nursing home or living in the community with full-time homecare were excluded from the study. 75 participants living independently in the community or in senior housing without full-time homecare and/or skilled medical/nursing care were included in the study. These participants were selected since they may represent a model for successfully aging. Of those 75, 7 participants were further excluded from the analyses due to their missing responses. Thus, 68 participants (ages 95-106y) were included in the analyses of this study. Details of the study's sampling and recruitment have been described previously [29, 48]. Ashkenazi Jews have been a well-studied group for the purposes of gene mapping as well as identifying genetic and other factors associated with exceptional longevity due to their genetic as well as socio-cultural homogeneity, although their survival and causes of death are similar to those reported for the majority U.S. population [48].

### Procedure

Detailed study procedures have been described previously [29, 48]. A single research nurse visited centenarian participants in their homes to obtain a medical history and administer the MMSE. The Centenarian Questionnaire [49] which includes the Personality Outlook Profile Scale (POPS) [19] was distributed in person with an instruction to each participant who was asked to complete it with the assistance of their adult children if necessary. The assistance of informants was used to maximize reliability due to some centenarians’ physical, sensory, and cognitive difficulties filling out the questionnaire. Adequate levels of personality rating agreements between self-reports and proxy-informant reports (e.g., family members) have been reported in other centenarian studies [46, 47].

### Measures

#### The Centenarian Questionnaire

The structured 98-item questionnaire, developed by the Longevity Genes Project at Albert Einstein College of Medicine, measures participants’ personal demographics, personality characteristics, health/medical history, self-rated health, and health-related behaviors [19, 50]. Participants’ current self-rated health was assessed by a question, “how would you rate your health presently?” with four choices of responses including “Very Good, Good, Average, and Not Good.” History of medical illnesses was assessed by participants’ affirmative responses to questions about if they ever had cancer, heart attack, stroke/transient ischemic attack, and/or diabetes. These illnesses were selected according to the data on leading causes of death in the U.S. [49]. The composite score of participants’ history of medical illnesses was computed by the summation of participants’ affirmative responses to questions about whether they had these illnesses.

#### The Personality Outlook Profile Scale (POPS)

This is a brief self- and informant-report measure of personality which represents personality phenotypes which may contribute to successful aging. POPS consists of two domains and six items: Positive Attitude Towards Life (PATL: optimism, easygoing, laughter, and introversion/ outgoing) and Emotional Expression (EE: expressing emotions openly and not bottling up emotions). The Centenarian Questionnaire served as the basis for the development of this measure. Items were developed using a rational content-based approach and derived from extant longevity research and theoretical concepts. They reflected centenarians’ lifetime personality and related behaviors. Two robust domains consisting of six items, PATL and EE, were selected from original eleven items. A composite score of each domain was creased based on a mean z-score of respective items for each domain. Higher scores reflect more favorable personality characteristics. In our previous study, these domains showed adequate internal consistency (α =.65 for PATL and α =.63 for EE), and this measure was cross-validated with the NEO-Five Factor Inventory and Life Orientation-Test in self- and informant-report groups [19].

### Mini Mental Status Exam (MMSE)

This is a brief measure of cognitive status in adults and older adults. The MMSE is a 30-item measure which tests an individual's orientation, attention, calculation, recall, language, and motor skills at a given point in time. It is used to screen for cognitive impairment, estimate the severity of cognitive impairment, and follow the course of cognitive changes over time. A cutoff score of 24 has been recommended for the detection of dementia [50]. For centenarians, a lower cutoff score of 21 due to normal age-associated cognitive declines has been suggested for the detection of dementia [39-41]. The internal consistency of the MMSE has been reported to be between .82 and .84 in older adults admitted to a medical service. The test-retest reliability in people with dementia ranged from .75 to .94. The MMSE has also been reported to have adequate levels of concurrent validity with other measures of cognition and dementia [51-53]. In the present study, if a participant was unable to respond to an item due to any sensory deficits (e.g., visual/hearing loss, paralysis of limbs), an extrapolated score was computed by multiplying the obtained score by 30, and dividing by the maximum obtainable score (the possible sum score for all the items that the participant attempted) [53].

### Statistical Methods

Analyses were completed with the statistical software, SPSS Statistics (Version 17.0). Multiple regression analyses were used to evaluate the relationships of the POPS to self-rated health and MMSE scores as well as the relationship between self-rated health and MMSE scores. The hierarchical regression analyses were conducted to adjust for the effects of demographic variables including age, gender, marital status, and years of education and history of medical illnesses on MMSE scores. In an exploratory analysis using Stata Version 12.1, structural equations modeling was used to determine the mediating role of PATL on the relationship between self-rated health and cognitive function.
